# Risk factors for partial placental retention after Cesarean delivery: a preliminary report

**DOI:** 10.1002/uog.70204

**Published:** 2026-04-09

**Authors:** L. Berg, M. Igielman, D. Jurkovic, E. Jauniaux

**Affiliations:** ^1^ EGA Institute for Women's Health, Faculty of Population Health Sciences, University College London (UCL) London UK

**Keywords:** assisted reproductive technology (ART), Cesarean delivery, hemorrhage, retained placenta, risk factors, ultrasound imaging, uterine anomaly

## Abstract

**Objective:**

To identify risk factors for partial placental retention (PPR) after Cesarean delivery (CD).

**Methods:**

This was a retrospective case–control study of patients with suspected complications after CD, including 25 cases of PPR and 75 controls without evidence of PPR. The diagnosis of PPR was made using high‐resolution ultrasound and was confirmed histologically in all cases. To identify potential risk factors for PPR, we compared demographic and clinical data between cases of PPR and controls.

**Results:**

Mode of conception, congenital uterine anomaly (CUA) and clinical indication for postpartum ultrasound assessment were all significantly associated with PPR after CD on univariable analysis. Patients with PPR were more likely to have conceived using assisted reproductive technology (9/22 (40.9%) *vs* 13/75 (17.3%); odds ratio (OR), 3.30 (95% CI, 1.17–9.33); *P* = 0.02), have a CUA (3/25 (12.0%) *vs* 1/75 (1.3%); OR, 10.1 (95% CI, 1.00–101.93); *P* = 0.05), and present with prolonged postpartum bleeding as the main indication for ultrasound assessment (20/25 (80.0%) *vs* 25/75 (33.3%)) as compared to acute bleeding (3/25 (12.0%) *vs* 7/75 (9.3%); OR, 0.54 (95% CI, 0.12–2.34)) or other indications (2/25 (8.0%) *vs* 43/75 (57.3%); OR, 0.06 (95% CI, 0.01–0.27)) (*P* < 0.001). Both indication for ultrasound assessment and presence of a CUA were retained in the multivariable analysis. In 19/20 (95.0%) cases with PPR for which data on placental location were available, the placenta was located in the upper uterine cavity antenatally, and none of the patients with PPR had antenatal ultrasound signs suggestive of a high probability of placenta accreta spectrum at birth. In all cases of PPR, the retained placental tissue was removed entirely using polyp or ovum forceps under ultrasound guidance, indicating that the placenta was not abnormally attached to the myometrium.

**Conclusions:**

Patients with a known CUA should be advised that they are at higher risk of PPR, and the obstetric team should take measures to minimize this risk. Prolonged postpartum bleeding is predictive of PPR after CD, and patients experiencing this should be referred for ultrasound assessment without delay. PPR after CD is not diagnostic of placenta accreta spectrum and surgical evacuation of the uterus is unlikely to be complicated. © 2026 The Author(s). *Ultrasound in Obstetrics & Gynecology* published by John Wiley & Sons Ltd on behalf of International Society of Ultrasound in Obstetrics and Gynecology.

## INTRODUCTION

Primary placental retention, defined as failure of expulsion of the placenta within 30 min of birth with active management or within 60 min of birth with physiological management^1^, is a clinical diagnosis made during the third stage of labor. Retention of the entire placenta after delivery of the fetus causes rapid bleeding. As such, primary placental retention is the second‐leading cause of postpartum hemorrhage (PPH) after uterine atony and, since placental retention prevents physiological postpartum myometrial contraction, these two conditions are linked^2^. Owing to variability in diagnostic criteria, access to bedside ultrasound and management protocols, the estimated prevalence of primary placental retention varies from 0.5% to 4.8% of births^2^, ^3^, ^4^.

Partial placental retention (PPR) is defined as retention of fragments of placental villous tissue after the main body of the placenta has been delivered^5^, ^6^, ^7^. PPR is an ultrasound diagnosis that may be made days, weeks or months after birth. The severity of clinical presentation varies from mild prolonged bleeding to acute sepsis and hemorrhage. Management is usually uterine evacuation.

PPR is a common complication of pregnancy loss before 20 weeks' gestation^8^, ^9^. PPR after third‐trimester vaginal birth is less common and has been linked to third stage labor complications^5^, ^6^  and to placenta accreta spectrum (PAS). PPR after Cesarean delivery (CD) has not been commonly described, despite the fact that in a previous multicenter study, one‐fifth of cases of PPR following third‐trimester births occurred after CD^7^.

The mechanism of placental delivery is different for CD compared with vaginal birth. During CD, the completeness of placental removal is checked by the surgeon exploring the uterine cavity, rather than relying solely on inspection of the placenta. In view of this, the risk factors for PPR after CD are also likely to be different. Ultrasound is used routinely to identify retained tissue after miscarriage and delivery^10^ but data on the role of ultrasound imaging in the diagnosis and management of PPR are limited. The aims of this study were to assess risk factors for PPR after CD and to evaluate the role of ultrasound imaging in its diagnosis.

## METHODS

### Study design and participants

We conducted a retrospective case–control study of consecutive patients with suspected complications following CD between January 2010 and January 2025 at the Gynaecology Diagnostic and Outpatient Treatment Unit, University College Hospital, London, UK. We identified cases of PPR and compared them with a control group of patients who were referred with suspected postoperative complications but did not have evidence of PPR on ultrasound assessment. We selected three controls for each case of PPR.

CD was performed following UK national guidance, which recommends that the placenta is delivered using controlled cord traction (CCT) with or without external compression of the uterine fundus^11^ with routine administration of a uterotonic (100 μg of intravenous carbetocin, or 5 IU of intravenous oxytocin if carbetocin is contraindicated) to initiate and maintain adequate uterine contractility^12^. Manual delivery of the placenta was performed if CCT failed. Following delivery of the placenta, a manual check of the uterine cavity, with or without a swab, was performed.

Demographic data (age, body mass index (BMI), gravidity, parity, ethnicity), previous obstetric and gynecological history, pre‐existing uterine conditions or surgery (operative hysteroscopy, surgical management of miscarriage, surgical termination of pregnancy, CD and myomectomy), mode of conception, antenatal placental location, delivery information and postpartum symptoms (including abnormal bleeding, pelvic pain and signs of sepsis) were collected. All ultrasound data and images were recorded and stored in a specialized database (ViewPoint Version 5; Bildverargeritung GmbH, Munich, Germany). For the purpose of analyzing the primary indication for ultrasound assessment, patients were divided into three groups: those with prolonged postpartum bleeding (vaginal bleeding similar to or less than a period, persisting for more than 4 weeks after birth), those with acute bleeding (sudden‐onset, heavy vaginal bleeding requiring emergency hospital admission) or those assessed for any other indication, including pelvic pain, suspected sepsis or concern of the operating team. PPH and major obstetric hemorrhage were defined as estimated blood loss of 1000 to < 1500 mL and ≥ 1500 mL during CD, respectively.

Surgical uterine evacuation was carried out in an outpatient setting under local anesthesia or as a day‐case procedure under general anesthesia. The procedure was performed in the outpatient setting if the patient was motivated to avoid general anesthesia and good views of the uterine cavity could be obtained with transabdominal sonographic (TAS) guidance. If performed in the outpatient setting, cervical ripening was achieved with a single synthetic hydrogel rod (Dilapan‐S®; Medicem Technology, Kamenné Žehrovice, Czech Republic) inserted to the level of the internal cervical os under local anesthetic and removed after 4–6 h. All uterine evacuation procedures were performed using polyp or ovum forceps to remove tissue in a targeted fashion under continuous TAS guidance. If performed under general anesthesia and TAS views were suboptimal, continuous transrectal ultrasound was performed. Sharp curettage was avoided. The uterine content was sent for histological examination. Only cases in which villous tissue was confirmed histologically were included in the analysis.

Ethical committee approval was obtained prior to the start of this study (National Health Service Health Research Authority: 18/WM/0328). Retrospective patient consent was waived, as all records were examined in the unit in which the procedure was undertaken and all data were fully anonymized.

### Ultrasound protocol

**Figure 1:**
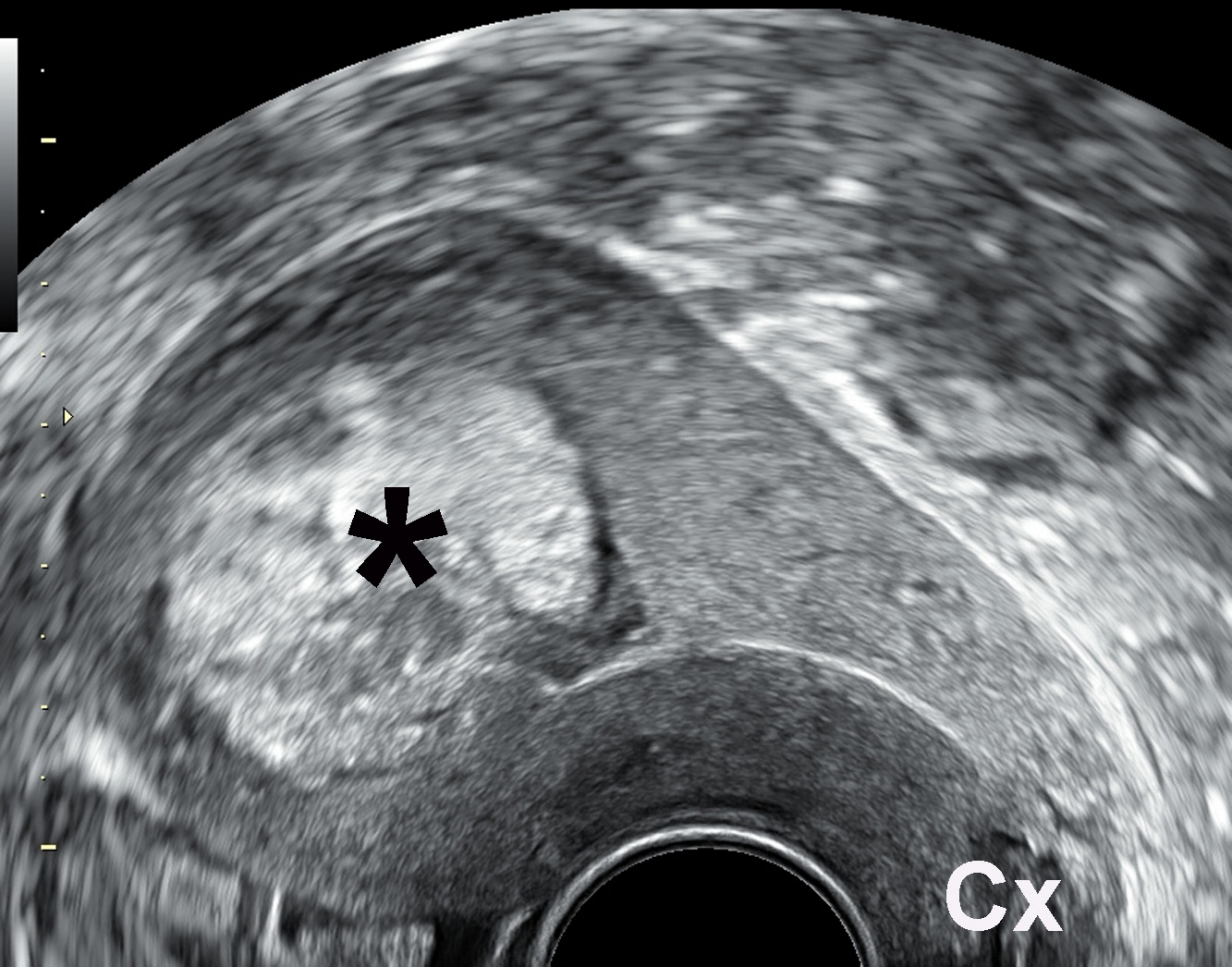
Grayscale transvaginal ultrasound image showing retained placental tissue (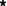) in a patient presenting with vaginal bleeding 4 weeks after Cesarean delivery. Cx, cervix.

**Figure 2:**
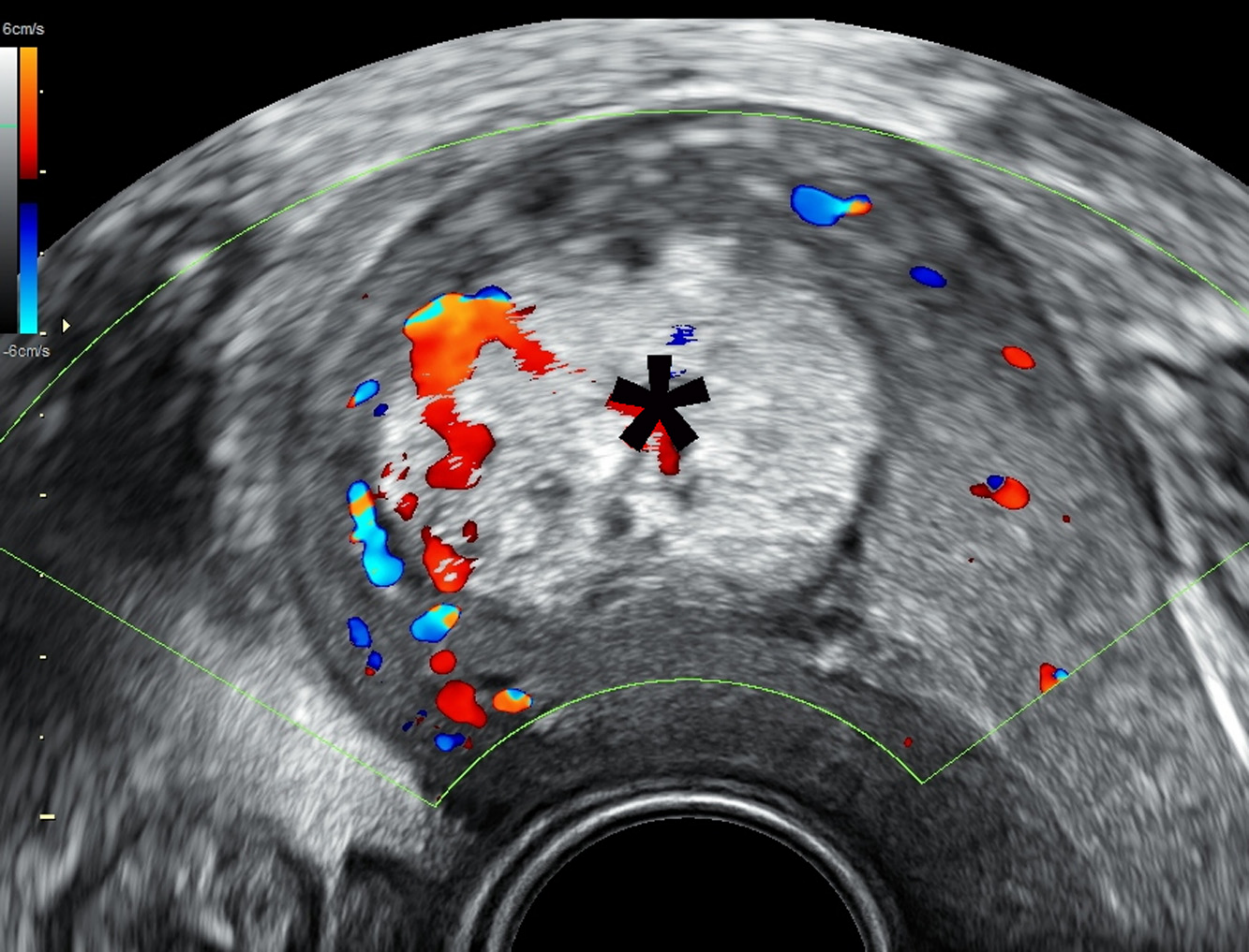
Color Doppler image mapping of retained placental tissue (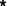) and the surrounding uterine circulation in a patient presenting with vaginal bleeding 4 weeks after Cesarean delivery.

All ultrasound examinations for PPR were performed by TAS and/or transvaginal sonography (TVS) using high‐resolution ultrasound equipment (Voluson 730 and Voluson E8 Expert; GE Medical Systems, Milwaukee, WI, USA) with color Doppler imaging (CDI) to assess uteroplacental blood flow. The presence of uteroplacental blood flow was defined as a color score of 2 or more on CDI^13^. All examinations during the study period were performed by clinical fellows (Level‐II operators) under the supervision of a team of experienced (Level‐III) operators^14^. An ultrasound diagnosis of PPR was made when the uterine cavity contained characteristic echogenic content (Figure 1), corresponding to the location of the placenta in the antenatal period, with or without vascularity on CDI (Figure 2). When identified, retained placental tissue was measured in three orthogonal planes and the mean diameter was calculated. Uterine morphology was assessed by examining the uterus in the transverse plane to evaluate division of the fundal aspect of the cavity and the presence of both interstitial tubes. If the interstitial portion of the Fallopian tube was absent, a diagnosis of unicornuate uterus was made. In patients with evidence of fundal indentation, three‐dimensional ultrasound was used to differentiate between arcuate, subseptate and bicornuate uterus, according to the American Society for Reproductive Medicine criteria^15^.

### Statistical analysis

Mean ± SD was calculated for normally distributed continuous variables and median (interquartile range (IQR)) was calculated for continuous variables that were not normally distributed. Categorical variables were presented as *n* (%). Baseline characteristics of the groups were compared using the unpaired *t*‐test for normally distributed continuous variables, the Mann–Whitney *U*‐test for continuous variables that were not normally distributed and the chi‐square test for categorical variables. The main analysis examined risk factors associated with PPR. Owing to the binary nature of the outcome, analysis of all risk factors was performed using logistic regression. The association between each risk factor of interest and the presence of PPR was examined separately in a series of univariable analyses. Subsequently, the joint association between the risk factors and the presence of PPR was assessed in multivariable analysis. To restrict the number of variables in this second stage of the analysis, only risk factors with a univariable *P*‐value of < 0.1 were included. A backwards selection procedure was used to retain only the significant variables in the final model. Data analysis was performed using SPSS version 28.0.1.1 (IBM Corp., Armonk, NY, USA). *P* < 0.05 was considered statistically significant.

## RESULTS

### Baseline characteristics

We reviewed the records of 25 consecutive patients with an ultrasound and histological diagnosis of PPR after CD. The control group comprised 75 consecutive patients who were referred with suspected complications of CD during the same period, in whom ultrasound examination excluded PPR.

Baseline characteristics of the 25 patients with PPR and 75 controls are shown in Table 1. Cases with PPR were significantly older than controls (*P* = 0.02) and more likely to have conceived via assisted reproductive technology (ART) (*P* = 0.02). All patients who conceived via ART had undergone *in‐vitro* fertilization. There were no differences in parity, BMI or ethnicity between the two groups.

**Table 1:** Baseline characteristics and indications for ultrasound assessment of patients with partial placental retention after Cesarean delivery (cases) *vs* those without (controls)

Characteristic	Cases (*n* = 25)	Controls (*n* = 75)	*P*
Maternal age (years)	36.8 ± 5.4	33.7 ± 5.8	0.02
Gravidity			0.76
1	9/24 (37.5)	23 (30.7)	
2	9/24 (37.5)	28 (37.3)	
≥ 3	6/24 (25.0)	24 (32.0)	
Parity			0.09
0	12/24 (50.0)	41 (54.7)	
1	11/24 (45.8)	20 (26.7)	
≥ 2	1/24 (4.2)	14 (18.7)	
BMI (kg/m^2^)	24.0 (22.0–27.0)	25 (22.5–29.0)	0.20
Ethnicity			0.22
White	16/23 (69.6)	29/64 (45.3)	
Black	3/23 (13.0)	10/64 (15.6)	
Asian	2/23 (8.7)	15/64 (23.4)	
Mixed/other	2/23 (8.7)	10/64 (15.6)	
Mode of conception			0.02
Spontaneous	13/22 (59.1)	62 (82.7)	
ART	9/22 (40.9)	13 (17.3)	
Indication for ultrasound assessment			< 0.001
Prolonged postpartum bleeding	20 (80.0)	25 (33.3)	
Acute bleeding	3 (12.0)	7 (9.3)	
Suspected sepsis	2 (8.0)	20 (26.7)	
Pelvic pain	0 (0)	12 (16.0)	
Concerns of the operating team	0 (0)	9 (12.0)	
Concerns on clinical examination	0 (0)	1 (1.3)	
Patient request	0 (0)	1 (1.3)	

Data are given as mean ± SD, *n/N* (%), median (interquartile range) or *n* (%). ART, assisted reproductive technology; BMI, body mass index.

### Ultrasound findings

The indications for ultrasound assessment are shown in Table 1. The median diameter of retained placental tissue visualized on ultrasound assessment was 22 (IQR, 16–28) mm. Uteroplacental vascularity was demonstrable on CDI in 14/25 (56.0%) PPR cases. In all cases, the retained placental tissue was located in the upper uterine cavity and the myometrium around the placental tissue appeared structurally normal on TVS and TAS. In 19/20 (95.0%) cases in which data on antenatal placental location were available, the placenta was located in the upper part of the uterine cavity and there were no ultrasound signs suggestive of PAS^16^. Overall, 3/25 (12.0%) cases had a congenital uterine anomaly (CUA), including two with a unicornuate uterus (one of whom had undergone previous excision of a rudimentary horn) and one with a bicornuate uterus. In the two cases of PPR with a unicornuate uterus, the placental location was posterior high and anterior high, respectively. In the case of PPR with a bicornuate uterus, the placental location was posterior high within the right uterine horn. One control was diagnosed with a subseptate uterus. In all cases of PPR, the retained placental tissue was removed entirely using polyp or ovum forceps under ultrasound guidance.

### Clinical variables

A summary of the univariable analysis examining the separate association between possible risk factors and PPR is shown in Table 2. Patients with PPR, compared with controls, had significantly higher odds of having a CUA (3/25 (12.0%) *vs* 1/75 (1.3%); odds ratio (OR), 10.1 (95% CI, 1.00–101.93); *P* = 0.05) and having conceived via ART (9/22 (40.9%) *vs* 13/75 (17.3%); OR, 3.30 (95% CI, 1.17–9.33); *P* = 0.02). Patients with PPR were also more likely to present with prolonged postpartum bleeding as their main indication for ultrasound assessment (20/25 (80.0%) *vs* 25/75 (33.3%)), as compared with acute bleeding (3/25 (12.0%) *vs* 7/75 (9.3%); OR, 0.54 (95% CI, 0.12–2.34)) or other concerns (2/25 (8.0%) *vs* 43/75 (57.3%); OR, 0.06 (95% CI, 0.01–0.27)) (*P* < 0.001).

**Table 2:** Univariable analysis of association between risk factors and partial placental retention after Cesarean delivery (CD)

Variable	Cases (*n* = 25)	Controls (*n* = 75)	OR (95% CI)	*P*
Maternal age				0.21
< 35 years	10 (40.0)	41 (54.7)	1	
≥ 35 years	15 (60.0)	34 (45.3)	1.81 (0.72–4.54)	
BMI				0.19
Healthy	14/23 (60.9)	29/72 (40.3)	1	
Underweight	1/23 (4.3)	8/72 (11.1)	0.26 (0.03–2.28)	
Overweight	8/23 (34.8)	35/72 (48.6)	0.47 (0.17–1.28)	
Gestation				0.40
Singleton	22 (88.0)	70 (93.3)	1	
Multiple	3 (12.0)	5 (6.7)	1.91 (0.42–8.64)	
Previous pregnancy				0.53
No	9/24 (37.5)	23 (30.7)	1	
Yes	15/24 (62.5)	52 (69.3)	0.74 (0.28–1.93)	
Previous birth				0.69
No	12/24 (50.0)	41 (54.7)	1	
Yes	12/24 (50.0)	34 (45.3)	1.21 (0.48–3.03)	
Previous uterine surgery				0.85
No	13/24 (54.2)	39 (52.0)	1	
Yes	11/24 (45.8)	36 (48.0)	0.92 (0.36–2.30)	
Congenital uterine anomaly				0.05
No	22 (88.0)	74 (98.7)	1	
Yes	3 (12.0)	1 (1.3)	10.1 (1.00–101.93)	
Conception				0.02
Spontaneous	13/22 (59.1)	62 (82.7)	1	
ART	9/22 (40.9)	13 (17.3)	3.30 (1.17–9.33)	
Placental location				0.89
High	19/20 (95.0)	67/70 (95.7)	1	
Low	1/20 (5.0)	3/70 (4.3)	1.18 (0.12–11.96)	
Gestational age at delivery				0.57
Term	18/22 (81.8)	54/71 (76.1)	1	
Preterm	4/22 (18.2)	17/71 (23.9)	0.71 (0.21–2.37)	
Type of CD				0.18
Elective	12/24 (50.0)	26 (34.7)	1	
Emergency	12/24 (50.0)	49 (65.3)	0.53 (0.21–1.35)	
Grade of surgeon				0.39
Consultant	7/15 (46.7)	24/69 (34.8)	1	
Resident doctor	8/15 (53.3)	45/69 (65.2)	0.61 (0.20–1.88)	
Intraoperative blood loss				0.57
No	14/21 (66.7)	54/71 (76.1)	1	
PPH*	3/21 (14.3)	5/71 (7.0)	2.31 (0.49–10.87)	
MOH†	4/21 (19.0)	12/71 (16.9)	1.29 (0.36–4.60)	
Blood transfusion				0.39
No	18/19 (94.7)	64/73 (87.7)	1	
Yes	1/19 (5.3)	9/73 (12.3)	0.40 (0.05–3.33)	
Indication for ultrasound assessment				<0.001
Prolonged postpartum bleeding	20 (80.0)	25 (33.3)	1	
Acute bleeding	3 (12.0)	7 (9.3)	0.54 (0.12–2.34)	
Other‡	2 (8.0)	43 (57.3)	0.06 (0.01–0.27)	

Data are given as *n* (%) or *n*/*N* (%).

* Postpartum hemorrhage (PPH) defined as estimated blood loss of 1000 to < 1500 mL during CD.

† Major obstetric hemorrhage (MOH) defined as estimated blood loss ≥ 1500 mL during CD.

‡ Other indications include suspected sepsis, pelvic pain, concerns of the operating team, concerns on clinical examination and patient request. ART, assisted reproductive technology; BMI, body mass index; OR, odds ratio.

A summary of the multivariable analysis examining the association between possible risk factors and PPR is presented in Table 3. This analysis showed that the indication for ultrasound assessment and the presence of a CUA were predictors of PPR after CD. The results for indication for ultrasound examination were highly significant (*P* = 0.002) while the results for CUA did not reach statistical significance (*P* = 0.09). However, owing to the large effect of CUA (the odds of PPR were almost 12 times higher for patients with CUA compared with the odds of those without), this factor was retained in the final model. After adjusting for the effects of these two variables, there was no additional effect of method of conception, which had been significant in the univariable analyses.

**Table 3:** Multivariable analysis of association between risk factors and partial placental retention after Cesarean delivery

Variable	Odds ratio (95% CI)	*P*
Congenital uterine anomaly		0.09
No	1	
Yes	11.9 (0.68–207.21)	
Indication for ultrasound assessment		0.002
Prolonged postpartum bleeding	1	
Acute bleeding	0.61 (0.14–2.69)	
Other	0.06 (0.01–0.28)	

## DISCUSSION

### Main findings

This study has identified a potential link between CUA and PPR and shown that prolonged postpartum bleeding is the most significant predictive factor for PPR following CD. Our findings emphasize that PPR is a potential complication of CD and highlight the importance of investigating postpartum patients who are concerned about vaginal bleeding, regardless of mode of delivery.

### Strengths and limitations

To our knowledge, this is the first study to investigate the risk factors for PPR after CD. In all cases, the ultrasound diagnosis of PPR was confirmed histologically. The use of controls with similar symptoms allowed us to identify which clinical presentation was most associated with PPR. The main limitation of this study is its retrospective design, which may have resulted in incomplete data, particularly regarding the suspicion of PAS. Suspicion of PAS may be mentioned to patients by the obstetric team or in the referral for ultrasound assessment but not recorded in medical notes, potentially underestimating this indication for ultrasound examination. Additionally, we were not able to match cases and controls for all baseline characteristics owing to the low number of patients who undergo a postpartum ultrasound assessment. Finally, this study was carried out at an advanced tertiary gynecological unit with a high level of experience in diagnostic ultrasound and invasive procedures under ultrasound guidance, which may limit the generalizability of the results.

### Comparison with other studies

This study challenges the traditional perception that PPR is mainly a complication of vaginal birth. Our study did not replicate the association of PPR with PPH and blood transfusion identified in previous studies^5^. The association between PPR and high blood loss at delivery may be more significant at vaginal birth, when, in the absence of surgical trauma to the uterus, a prolonged third stage due to PPR may be a stronger determinant of overall blood loss. Alternatively, high blood loss at delivery may be over‐represented in the control group in this study, who were referred for ultrasound examination owing to suspicion of complications. Larger studies including both vaginal and Cesarean births would be useful to confirm whether risk factors for PPR differ depending on mode of delivery.

Postpartum ultrasound is essential in the diagnosis and treatment of PPR, and those who experience persistent bleeding after CD should be promptly assessed. A previous study proposed categorization of patients with suspected PPR into high‐, medium‐ or low‐risk groups depending on the presence of echogenic uterine content with vascularity, the presence of echogenic uterine content without vascularity or the absence of characteristic uterine content on ultrasound^17^. Patients at high risk for PPR were managed with a follow‐up examination 10–14 days after the initial ultrasound examination and then surgically with hysteroscopy, if indicated, whereas those at medium or low risk for PPR were managed expectantly with a follow‐up examination at the end of the puerperium. In our center, we similarly consider characteristic echogenic uterine content with vascularity diagnostic, and we adopt a conservative approach for non‐diagnostic scans. However, we also diagnose and actively manage patients in whom there is no uteroplacental blood flow on CDI, assuming the echogenic uterine content appears characteristic of PPR. Indeed, 44% of histologically confirmed cases of PPR in this study did not have vascularity on CDI. Additionally, the previous protocol^17^ referred patients at high risk for PPR for hysteroscopic treatment, whereas our routine practice is ultrasound‐guided evacuation.

Pregnancies conceived using ART had a significantly higher risk of PPR after CD in the univariable analysis but not after adjustment for other factors in the multivariable analysis. Conception by ART has been associated with an increased risk of manual removal of the placenta following vaginal birth^18^ and with a higher incidence of placental shape anomalies^19^, which can lead to the retention of one or more placental lobes at vaginal delivery^19^, ^20^. Succenturiate lobes would also be more likely to be retained after the main body of the placenta is delivered via CD, and their absence is harder to detect when checking the placenta is complete.

In the last two decades, retrospective cohort studies have begun to classify clinical features previously associated with PPH, primary placental retention and uterine atony (such as difficult or piecemeal manual removal of the placenta, absence of spontaneous placental separation within 30 min after delivery in active management of the third stage, and heavy bleeding from the placentation site after removal of placenta previa) to suggest PAS^21^. Several authors have now included patients with PPR requiring uterine evacuation after vaginal birth in a cohort of patients with PAS, implying that ultrasound imaging can identify PAS after delivery^22^, ^23^, ^24^. In 95% of cases complicated by PPR in this study, the placenta was located in the upper uterine cavity antenatally, and none of the included cases of PPR presented with ultrasound signs associated with a high probability of PAS at birth^16^. In all cases, it was possible to remove the retained placental tissue under ultrasound guidance using forceps, confirming that the placenta was not abnormally attached.

### Future perspectives

CUAs have been associated with an increased risk of primary placental retention at vaginal birth. A recent systematic review and meta‐analysis showed a 70% increase (OR, 1.71 (95% CI, 1.16–2.52)) in the corresponding risk^25^. Our data suggest that some CUAs, such as unicornuate or bicornuate uterus, may also be risk factors for PPR. If part of the placenta develops within a uterine horn, delivering the placenta whole may be difficult and retained placental tissue may not be visible to the surgical team. Patients with CUA could be informed of this potential risk and counseled to monitor their lochia and maintain a low threshold to present with any concerns. Further multicenter studies would be useful to confirm this potential association.

### Conclusions

Prolonged postpartum bleeding is predictive of PPR after CD. A low threshold for ultrasound assessment in any patient presenting with concerns about vaginal bleeding is critical. Ultrasound diagnosis using B‐mode imaging is reliable but CDI is of limited value, as blood flow was absent in nearly half of the cases of PPR. Our findings suggest that patients diagnosed with CUA may be at increased risk of PPR after CD.

## Data Availability

The data that support the findings of this study are available from the corresponding author upon reasonable request.
